# The formal care costs of dementia: a longitudinal study using Swedish register data

**DOI:** 10.1007/s10198-024-01707-w

**Published:** 2024-07-30

**Authors:** Jennifer Zilling, Ulf-G. Gerdtham, Johan Jarl, Sanjib Saha, Sofie Persson

**Affiliations:** 1 Health Economics Unit, Department of Clinical Sciences (Malmö), Forum Medicum, Lund University, Sölvegatan 19, 223 62 Lund, Sweden; 2 Department of Economics, School of Economics and Management, Lund University, Scheelevägen 15B, 223 63 Lund, Sweden; 3 Centre for Economic Demography, Lund University, Scheelevägen 15B, 223 63 Lund, Sweden; 4 The Swedish Institute for Health Economics, Råbygatan 2, 223 61 Lund, Sweden

**Keywords:** Excess costs, Formal care, Dementia, Alzheimer’s disease, Register data, Sweden, I1

## Abstract

**Background:**

This study investigates the excess costs of dementia from healthcare, social care services, and prescription drugs 3 years before to 6 years after diagnosis. Further, sociodemographic cost differences are explored.

**Methods:**

Using Swedish register data from 2013 to 2016 to compare individuals diagnosed with dementia (n = 15,339) with population controls, the excess formal care costs for people with a dementia diagnosis are obtained with longitudinal regression analysis.

**Results:**

People with dementia incur higher formal care costs for all years studied compared to people without dementia. The excess costs vary from €3400 3 years before diagnosis to €49,700 6 years after diagnosis. The costs are mainly driven by institutional care, and solitary living is a strong predictor of high excess costs.

**Conclusion:**

The results show that the formal care costs of individuals with dementia are substantial, and that the economic burden of dementia in Sweden is larger than previously estimated.

**Supplementary Information:**

The online version contains supplementary material available at 10.1007/s10198-024-01707-w.

## Background

Dementia diseases stand among the costliest diseases and exceed the costs of cancer and heart disease combined [1, 2]. Global costs of dementia have been estimated to be US$1313 billion in 2019, or US$23,796 per person, corresponding to an increase in global costs by 62% since 2010 [3]. Exploring the European setting in a meta-analysis, Jönsson et al. estimate the annual cost per person in the Nordic countries to be €43,767 in 2021 prices [4]. Based on aggregated data, Wimo et al. present similar estimates (about €45,000) for the average societal cost per person with dementia in Sweden in 2012 [5]. The prevalence of dementia is expected to continue to increase due to an aging population and current health-related behaviours [6, 7]. Thus, reliable, and up-to-date estimates of the economic burden of dementia are necessary to provide decision-makers with the information needed to plan and allocate resources for future elder care.

Most studies investigating the costs of dementia use cross-sectional data and often lack a comparison group [8–14]. Thus, they do not capture the longitudinal development of costs over time, nor do they account for costs associated with normal aging. To address these limitations, Persson et al. and Frahm-Falkenberg et al. use longitudinal register data for their analyses, enabling a temporal perspective on excess costs in healthcare among people with dementia in southern Sweden and Denmark, respectively [15, 16]. Both studies find positive excess costs for healthcare up to 10 years before diagnosis, costs being more than twice as high in the dementia group in relation to the comparison group at the year of diagnosis, and that costs are substantially declining after diagnosis with no remaining excess cost 4–5  years after diagnosis. However, Persson et al. and Frahm-Falkenberg et al. do not study costs that occur in the social care sector, which has been found to account for the largest part of formal care costs after a dementia diagnosis [4, 5] and to increase substantially with worsened cognitive impairment [1, 4, 17–19].

The aim of this study is to investigate the excess costs from healthcare, social care services, and prescription drugs dispensed at pharmacies, which can be derived from dementia. Additionally, we aim to explore the distribution of these costs across different care sectors and over time, focusing on the 3 years before and 6 years after a formal diagnose of dementia. To do so we study the costs during the years 2013–2016 of people in southern Sweden who received a dementia diagnosis between 2010 and 2016 compared to matched population controls. Furthermore, we explore whether sociodemographic characteristics defined by sex, civil status, educational attainment, and foreign background are associated with cost differences in people with dementia. The results will provide a detailed and up-to-date picture of the economic burden of dementia and the potential benefits of preventing/postponing the progress of dementia, as well as to identify vulnerable subgroups within the dementia population and their informal caregivers. To our knowledge, this study represents the first comprehensive analysis of the full excess formal care costs related to dementia using Swedish register data.

## Methods

### Study setting and population

Sweden applies a decentralised Beveridge care system with publicly funded, and publicly and privately provided care. Twenty self-governed regions are responsible for providing adequate healthcare, while 290 autonomous municipalities manage social care services [20, 21]. Our study is restricted to the population living in the southernmost region of Sweden (Region Skåne) and its 33 municipalities due to the accessibility of primary care data that is not accessible on the national level. We consider the entire cost of formal care, and do not separate out patient fees.

### Data

The study population consists of people with a first dementia diagnosis during 2010–2016 obtained from Region Skåne’s Healthcare Utilisation Database. People with dementia is selected based on the ICD10 codes F00.0–F00.2, F00.9, G30.0, G30.1, G30.8, G30.9, F01.1–F01.3, F01.8–F02.0, F02.2–F02.4, F02.8, F03.9, F03–P, F10.6–7A, G32.0, and G31.8. A comparison group is constructed by matching (1:1) individuals without a dementia diagnosis before or during the study period, based on sex, age, and the municipality of residence at the time of diagnosis. This is done to ensure a relevant overlap between the case and control group in terms of background characteristics. Data from the National Patient Register is used to ensure that the individuals in the comparison group have not received a dementia diagnosis in another region. To this population, individual-level data from several registers have been linked by individual identification numbers.

Information on healthcare utilisation and associated costs for inpatient, outpatient, and primary care is obtained from Region Skåne’s Healthcare Utilisation Database. The Social Service Register (SSR) provided by the Swedish National Board of Health and Welfare (NBHW) gives information on municipal social care services [22]. Social care includes home care (e.g., personal care and support, and household chores), institutional care, short-term respite care, and day care activities (i.e., social activities and support to maintain mental and physical abilities). To calculate the costs for institutional care, home care, and short-term respite care, costs are collected from the open data source Kolada [23] and multiplied by the quantity of utilisation for the corresponding social care variable. The costs are the average cost per care recipient in Region Skåne for each studied type of care and year. The cost for day care is retrieved from the NBHW [24] and is based on the average cost in Region Skåne per granted person in 2012. Information on all prescribed drugs dispensed at pharmacies, including the costs, is provided by the NBHW in the National Prescribed Drug Register [25]. Sociodemographic information is retrieved from the Longitudinal Integrated Database for Health Insurance and Labour Market Studies (LISA) database [26].

All costs are adjusted to 2016 domestic consumer prices and converted to the average 2016 Euro exchange rate [27, 28]. The cost measures are presented in Table S1 in the supplementary materials. In cases where information on costs in the healthcare utilisation database and care utilisation in the Social Service Register is missing, imputation is carried out using several steps. These steps are presented in detail in the supplementary materials.

### Main variables

The main outcome variable in the analysis is the sum of all formal care costs, i.e., costs for healthcare, social care, and prescription drugs dispensed at pharmacies, that are linked to an individual in a year. The effect of main interest is that of a binary variable stating if the individual is diagnosed with dementia during the years 2010–2016.

The study period is 2013–2016 as the Social Service Register is only of sufficient quality in terms of collection frequency and consistency of information from the year 2013 [29]. Consequently, individuals can be observed for a maximum of 4 consecutive years, although due to the inclusion of individuals diagnosed in 2010–2016, they will contribute to different years in the time trend. For example, an individual diagnosed in the year of 2010 can be observed from 3 years after diagnosis (2013) to 6 years after diagnosis (2016) while an individual diagnosed in 2015 can be observed 2 years before and 1 year after diagnosis. With this strategy, a study period of 3 years before diagnosis to 6 years after diagnosis is covered, resulting in a total of 10 years for analysis. The year an individual dies is excluded so that each year reflects the costs for a full year alive.

### Statistical analysis

To predict the excess formal care costs over time, we employ an ordinary least squares regression. The regression model includes interaction variables between the binary variable indicating dementia and a categorical variable representing the number of years before and after diagnosis. Standard errors are clustered on the individual level to correct for the panel data structure. To prevent confounding we control for the following potential confounders that can have an impact on both the risk of dementia and the formal care costs: civil status (being married/cohabitant at the year of diagnosis), educational attainment, foreign background (defined as being born outside Sweden or having both parents born outside Sweden), number of children, sex, age at diagnosis (categorised as being younger than 70, 70–89 or 90 years and older), the Elixhauser comorbidity index based on the 5 years preceding diagnosis, municipality of residence in each given year, and fixed effects of calendar year. The matched study design does not require conditional analysis, as the matching is only used to create a representative control group [30].

Heterogeneity analyses are performed to investigate differences in costs by sex, civil status, education, and foreign background. The methodology used for these analyses follows the same setup as the main estimates, with the addition of a sociodemographic dimension to the interaction term. Moreover, a logistic regression model is used to predict the proportion in the dementia and comparison groups that have utilised home care and institutional care each year. This is to further investigate the underlying dynamics behind the potential cost drivers.

In cost data with limited samples, the assumption of normally distributed data is often violated. To validate the robustness of the results, we employ a generalized estimating equations model with unstructured correlation. All statistical analysis is performed using Stata version 17. Results are considered significant at the 5% level.

## Results

### Descriptive statistics

Table 1 shows summary statistics for the study population, consisting of 15,339 individuals diagnosed with dementia and their comparators. The mean age at diagnosis is 81 years and 41% of the sample are men. The people in the dementia group are less likely to have children, to be married or cohabiting, and to have obtained higher education, but more likely to have a foreign background. The Elixhauser comorbidity index indicates that those with dementia are in somewhat worse health compared to the comparison group in the 5 years before diagnosis, with mortality being twice as high in the dementia group over the study period.

**Table 1:** Population characteristics at diagnosis

	Dementia	Population control	${p}^{a}$
Number of individuals	15,339	15,339	1.000
Male, n (%)	6235 (40.7)	6235 (40.7)	1.000
Year of birth, mean (SD)	1933 (9.5)	1933 (9.5)	1.000
Year of diagnosis, mean (SD)	2013 (1.8)	2013 (1.8)^1^	1.000
Age at diagnosis, mean (SD)	81 (9.7)	81 (9.7)^1^	1.000
Highest educational degree, n (%)			
Compulsory	7407 (48.3)	7136 (46.5)	0.002
Upper secondary	5084 (33.1)	5241 (34.2)	0.058
Higher education	2443 (15.9)	2641 (17.2)	0.002
Missing	405 (2.6)	321 (2.1)	0.002
Foreign background, n (%)	2166 (14.1)	1797 (11.7)	0.000
Married or cohabiting, n (%)	4695 (30.6)	5677 (37.0)	0.000
Number of children, n (%)			
None	2437 (15.9)	2189 (14.3)	0.000
1–2	8486 (55.3)	8746 (57.0)	0.003
3–4	3839 (25.0)	3874 (25.3)	0.645
5 or more	577 (3.8)	530 (3.5)	0.150
Elixhauser comorbidity index, mean (SD)	2.3 (2.0)	2.0 (1.9)	0.000
Deceased at the end of study period (%)	5363 (35.0)	2424 (15.8)	0.000

SD is standard deviation and n is number of observations. ${p}^{a}$ is t-test and test of proportions for significant differences in the variables between the dementia group and the comparison group

^1^By design, the population controls are matched according to age and year of diagnosis although the individuals in this group never received a dementia diagnosis throughout the study period

### Main results

Table 2 presents the predicted total formal care costs per person for people with a dementia diagnosis and their comparators, as well as the number of observations of respective years. The full underlying regression output is available in the Supplementary Table S2. The costs are significantly higher for the dementia group across all observed years. Already 3 years before diagnosis, the dementia group incurs excess costs of €3410. The excess costs almost triple to €23,376 in the year of diagnosis, compared to €8022 the year before, and then continue to increase each year, reaching €49,727 6 years after diagnosis. The results remain robust when employing the generalized estimating equations model.

**Table 2:** Predicted formal care costs of people with dementia and population controls from 3 years before to 6 years after diagnosis, expressed in 2016 Euro prices

Years from diagnosis	Dementia			Population control			Difference	
	n	Mean	95% CI	n	Mean	95% CI	Euro	(%)
− 3	1760	8484	7146–9822	1760	5074	4078–6070	3410*	(67)
− 2	4339	12,310	11,366–13,253	4339	6856	6175–7537	5454*	(80)
− 1	7435	17,403	16,672–18,134	7435	9381	8801–9961	8022*	(86)
0	8911	34,454	33,560–35,347	8911	11,077	10,552–11,602	23,376*	(211)
1	8598	45,845	44,790–46,900	9444	11,740	11,228–12,251	34,105*	(291)
2	7399	51,917	50,758–53,075	8359	12,170	11,619–12,722	39,746*	(327)
3	5944	56,765	55,427–58,103	6885	12,889	12,304–13,475	43,876*	(340)
4	3672	59,662	58,026–61,297	4613	13,704	12,923–14,486	45,957*	(335)
5	1961	61,021	58,653–63,388	2700	13,978	12,903–15,054	47,042*	(337)
6	836	62,272	58,801–65,742	1192	12,545	11,155–13,935	49,727*	(396)

Adjusted for year, municipality, sex, age at diagnosis, education, foreign background, number of children, marital status and Elixhauser comorbidity index 5 years prior diagnosis. n is the number of observations

*Statistical significance at the 5% level. The percentage increase in costs due to dementia is calculated as the cost difference between people with dementia and population controls, divided by the costs for population controls

**Fig. 1:**
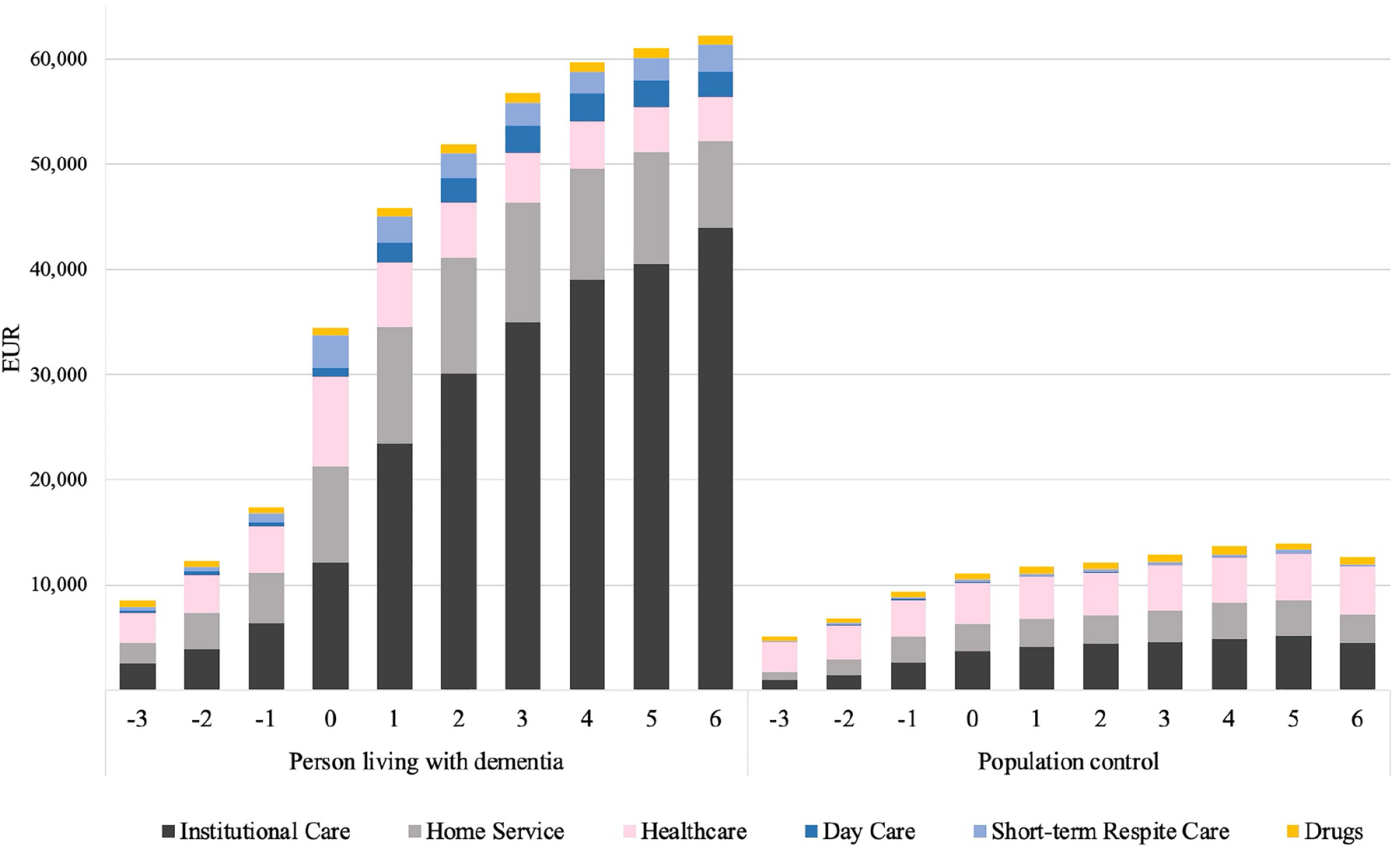
Predicted formal care costs of people with dementia and population controls from 3 years before to 6 years after diagnosis by care component. Costs are expressed in 2016 Euro prices

Figure 1 shows the predicted cost for each studied care component, while the detailed information is available in Table S3 in the supplementary materials. Institutional care constitutes the greatest share of the total cost in the dementia group during 1 to 6 years after diagnosis, ranging from 51% to 71% of total costs, corresponding to €23,452–€43,993. In the comparison group, institutional care never exceeds 37% of total costs or €5164. Furthermore, in the comparison group healthcare costs increase over time, and from 3 to 6 years after diagnosis, they align with those of individuals with dementia.

### Proportion receiving institutional- and home care

**Fig. 2:**
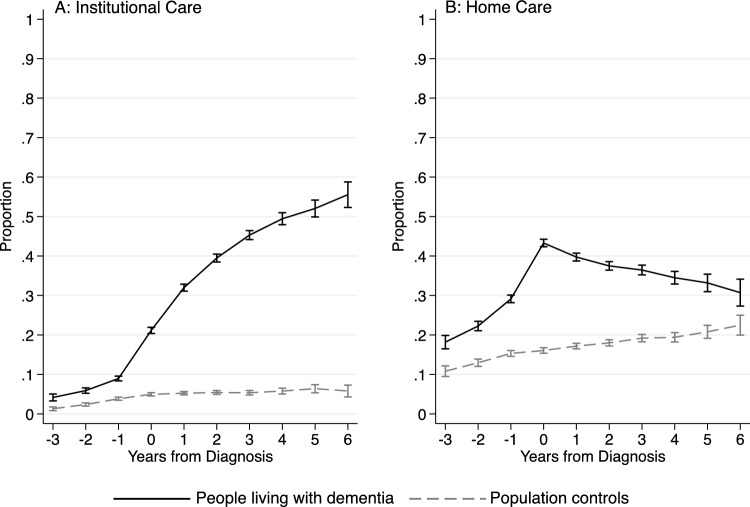
Predicted proportions receiving institutional care and home care services of people with dementia and population controls from 3 years before to 6 years after diagnosis. The whiskers indicate 95% confidence intervals

Figure 2 shows the predicted proportion of people receiving institutional- and home care for the dementia and comparison groups. In the dementia group, the proportion of individuals receiving institutional care increases from around 10% the year before diagnosis to over 20% in the year of diagnosis. Six years after diagnosis, the proportion receiving institutional care has increased to 56% in the dementia group. In the comparison group, the proportion with institutional care never exceeds 6%. The proportion of people receiving any amount of home care peaks at the year of diagnosis in the dementia population (43%) and subsequently declines to 31% 6 years after diagnosis. In the comparison group, the share receiving homecare never exceeds 23%.

### Heterogeneity analysis

**Fig. 3:**
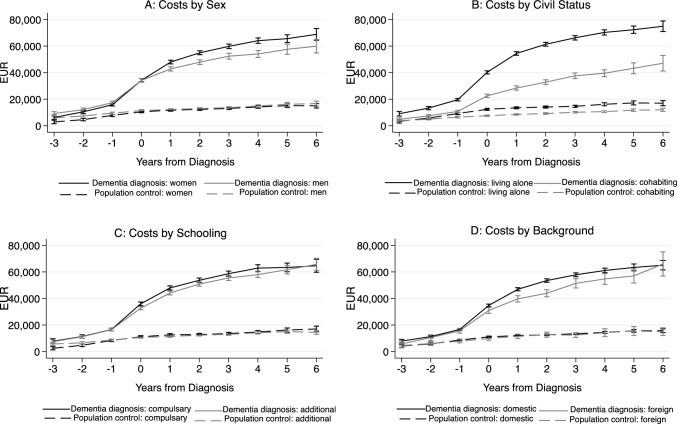
Predicted formal care costs by sex, schooling, background, and marital status of people with dementia and population controls from 3 years before to 6 years after diagnosis. Costs are expressed in 2016 Euro prices. The whiskers indicate 95% confidence intervals

Figure 3 shows the cost estimates by sex, civil status, level of education, and foreign background. After being diagnosed with dementia the costs are generally higher for women compared to men. Among people living with dementia, those of foreign background exhibit lower costs 1–3 years following diagnosis. Additional schooling, i.e. higher than compulsory, predicts slightly lower costs among people with dementia in the year of diagnosis and the year following diagnosis. Civil status is the largest cost predictor of people with dementia in the heterogeneity analysis. Those living with a partner at the time of diagnosis consistently incur lower costs compared to those living alone. Six years after diagnosis, people living with a partner at diagnosis still have lower costs compared to 1 year after diagnosis for people living alone, indicating a substantial cost reduction associated with living with a partner. This lower cost associated with living with a partner is also noted among the comparison group.

## Discussion

This study provides a detailed and up-to-date picture of the economic burden imposed by dementia, valuable for health economic simulation models assessing the cost-effectiveness of new treatment interventions over time. Dementia costs are distributed across different care sectors, and it is thus of importance to provide a comprehensive picture of the formal care costs. Our results show rising excess costs of formal care for individuals with dementia, from 3 years before diagnosis to 6 years after diagnosis, beginning at €3410 and increasing to €49,727. The excess costs we find are likely a combination of increased care utilization related directly to dementia and from comorbidities that may develop over time after diagnosis.

The study estimates show that excess formal care costs for individuals with dementia are significantly higher than the net cost estimate of approximately €29,000 for Sweden in 2012, provided by Wimo et al. [5]. This figure corresponds to about €27,000 in 2016 consumer prices, adjusted for the depreciation of the Swedish currency (authors’ calculations). Our study yields higher costs for all years after diagnosis, with estimates increasing from €34,105 to €49,727. The difference in estimated costs would likely have been even more pronounced if our study design had allowed for a longer observation period after diagnosis, considering the upward trend in costs over time. The reason for the difference in results is likely multifactorial and may, beside differences in methodological approaches, be explained by regional and time varying cost differences in the care sector.

Additionally, when considering the gross cost estimates for the dementia group, i.e., the cost estimates without subtracting the costs attributed to the comparison group, the estimates vary from €45,845 to €62,272 after diagnosis. Previous research finds corresponding estimates to be €32,035 in the Nordic countries (2021 prices) [4], €14,288 in Europe (2015 prices) [14], and US$11,994 globally or US$25,341 among high-income countries (2019 prices) [3]. In comparison to previous studies, the estimates in this study are substantially higher.

Our results for excess healthcare costs among people with a dementia diagnosis show a peak in the year of diagnosis, whereafter the costs for healthcare steadily decline. This aligns with the results observed by Persson et al. and Frahm-Falkenberg et al. [15, 16]. The peak in excess healthcare costs during the year of diagnosis is partly due to the costs associated with the diagnosis procedure. It is also likely that these costs arise from care sought for other conditions, whereupon detection of a dementia disease is picked up by healthcare professionals. However, unlike healthcare costs, the total excess costs for formal care continue to rise each year throughout the study period, with healthcare constituting a relatively small share of the total costs in subsequent years. This suggests that a dementia diagnosis increases access to social care services, reducing reliance on healthcare services. This result aligns with Wimo et al.’s findings, which show that social care services, and in particular institutional care, account for most of the costs associated with a dementia diagnosis [5]. This also agrees with Jönsson et al.’s results about higher costs associated with increased disease severity are driven by institutional care [4].

The heterogeneity analysis reveals cost differences among people with a dementia diagnosis based on sex, educational attainment, civil status, and foreign background. Specifically, cohabiting at the time of diagnosis is strongly associated with reduced costs in formal care for people with a dementia diagnosis. Moreover, living with a partner is associated with lower costs also in the comparison group.

Education has previously been shown to delay the time to institutional living in dementia [31]. However, our findings show modest differences in care costs by educational attainment. People of foreign background having lower costs could reflect cultural differences in expectations to care for family members or be due to greater obstacles in accessing or trusting formal care. This result resembles findings from Denmark where nursing home residency among people with dementia is less likely for people of foreign background [32].

Under the assumption that the need for care is not influenced by background or sex, the lower formal care costs observed for people of foreign background and men may indicate a higher reliance on informal care. This implies that the lower costs for these groups are potentially balanced by a disproportionate burden of informal care. Several studies find that informal care accounts for the largest proportion of costs associated with a dementia diagnosis, varying between 50 and 90% [17, 33–36]. Jönsson et al. find that informal care constitutes a smaller proportion of the costs in the Nordic countries and present estimates of informal care, caregiver time, and work loss corresponding to about 27% of total costs [4]. Given the importance of informal care, it is of interest to further investigate related costs and obstacles in access to formal care for potentially vulnerable sociodemographic groups.

Accessing various sources of register data is an important strength of this paper, enabling the evaluation of excess formal care costs of dementia at the individual-level. As the onset of dementia is not random, several potential confounding effects from socioeconomic and demographic factors are controlled for given the rich dataset. However, the risk of biased estimates due to potential unobserved factors that could impact the difference in costs between individuals with a dementia diagnosis and their comparators is a constant problem in the non-experimental data setting.

People with dementia in southern Sweden can be tracked on a more extensive level as dementia diagnoses from primary care data are available. The primary care data also give important information for complete healthcare costs that would have been lost with national-level data. Region Skåne constitutes 13.4% of the population in Sweden (2021 figures) [37], and the average age, the proportion of people aged 65 years or older, and the proportion with higher education correspond to levels of the national average [38]. Assuming that the population in southern Sweden is representative of Sweden as a whole, geographical cost differences may still occur due to the decentralised care provided on municipal and regional levels. However, Sweden has an institutional system of distributing a large share of the care burden on the community, and thus the results can arguably be generalised to the broader Swedish setting. Further, we expect that the relative weight of specific care components can be generalised across countries where social care is predominantly characterised by formal care. Following Jönsson et al.’s [4]. study on cost structures in dementia care in Europe, this would indicate generalisability to the Nordic countries, and potentially also to Western Europe, Eastern Europe and the Baltics. However, the results should be generalised with caution for countries characterised by a large share of informal social care, such as Southern Europe and the Brittish Isles.

Excluding the year an individual dies, and thus not providing information of costs in close connection to death is a limitation of this study. However, as we study annual costs, including individuals that do not account for a full year alive would bias the estimates. Furthermore, the annual cost development of dementia is examined over time. This should not be interpreted as costs associated with different disease stages. Instead, our estimates reflect the general economic burden in close connection to diagnosis and each year constitutes individuals that were diagnosed at different stages in the disease course. However, to study the cost development in relation to severity of cognitive impairment would contribute to more detailed information on the effect of slowing down disease progression on societal costs.

To our knowledge, there has been no major changes in the overall care concepts and structures of people with dementia in Sweden since 2016, with few new efficient pharmaceuticals or interventions in diagnostic procedures. However, in 2017 the Swedish national board of health and welfare released new national guidelines for the care of dementia which emphasised the importance of regular and structured follow-up of people with dementia to assess the person’s need for medical and psychosocial support [39]. The guidelines state that the recommended measures may increase costs for health and social services due to an increased need for resources, which implies that even higher dementia related costs could be expected in more recent years.

## Conclusion

This study shows that the costs of formal care associated with dementia are substantial, and that the societal burden of dementia in Sweden is larger than previously estimated. Integrating these findings into health economic modelling allows for more accurate assessments of costs and benefits. This information is vital for decision-makers in making informed choices regarding resource allocation and developing effective strategies for dementia care.

## Supplementary Information

Below is the link to the electronic supplementary material.

Supplementary file1 (DOCX 42 KB)

## Data Availability

Results are based on non-public microdata that are accessible under certain conditions for statistical and scientific research.
