# Supplementary material for: The formal care costs of dementia: a longitudinal study using Swedish register data
Source: Eur J Health Econ. 2024 Jul 30;26(3):353–61. doi: 10.1007/s10198-024-01707-w (PMC11937048; doi:10.1007/s10198-024-01707-w)
Supplement: Supplementary file 1 — Supplementary file1 (DOCX 42 KB) [file 10198_2024_1707_MOESM1_ESM.docx]

**Supplementary Material**

**Strategies for imputation**

1. **Imputation strategy for the social care variables**

From 2013 and onwards the social care variables are by design registered monthly. However, in practice, inconsistent reporting entails missing values. To handle this for the institutional care variable, we employ a strategy in which missing values are filled in with the value of the preceding reported month with a carry forward command until a new value is reported for the variable. We do the same strategy for earlier time points with missing information if the successive value states that the person did not use institutional care. We do this with the assumption that if a person is not living in a nursing home in one period, then that person was not living in a nursing home before that point either. Studying the Social Service Register, Meyer, Sandström and Modig suggest imputation as a reasonable strategy to erase shorter gaps in the register [29]. In accordance with this, we believe the flaws of imputing missing values based on our assumptions are outweighed by the benefits of accessing unique register data that will give us information on the associated costs of a dementia diagnosis from individual-level data. For home care, we use the strategy of filling in missing values with the preceding reported month unless the individual starts reporting utilisation of institutional care instead. We do no imputation for short-term respite care and day care as we believe it is a too strong assumption to say they are continual until the opposite is being reported.

1. **Imputation for healthcare variables**

Values for cost information were imputed when the information was missing in the healthcare utilisation database. The imputation was conducted in several steps and inpatient- and outpatient care were imputed separately and on a yearly basis. For outpatient care, imputation was further separated for visiting physicians and other healthcare personnel, and private care. When DRG-weights were available, multivariate normal regressions were performed. DRG-weights, days in inpatient care, and whether the care episode was planned or not were included in the somatic inpatient care regression. DRG-weights were generally missing when costs were missing for inpatient psychiatric care and patient hotel visits and thus a per diem cost was predicted based on the number of days and whether the psychiatric episode was planned. Remaining missing information for costs was imputed using the average cost of somatic care adjusted for length of stay. For outpatient and primary care, missing costs were imputed based on DRG-weights and if the episode had been planned or not. When DRG-weights were missing, the average cost per patient and sector was used for physical visits. Non-physical visits with missing costs were assumed to have zero cost. Remaining missing costs were imputed with the average cost per sector.

**Cost Measures**

| Table S1: Cost measures for social care services in 2016 Euro prices. | | | | |
| --- | --- | --- | --- | --- |
| Year | *Institutional Care*  *(Yearly Average)* | *Short-term Respite Care*  *(Daily Average)* | *Home Service*  *(Cost/hour)* | *Day Care*  *(2012 Average)* |
| *2013* | 87,775 | 317 | 48 | 33,721 |
| *2014* | 92,166 | 338 | 42 | 33,721 |
| *2015* | 88,002 | 319 | 45 | 33,721 |
| *2016* | 91,039 | 385 | 50 | 33,721 |
| **Note:** Day care costs are retrieved from NBHW (Socialstyrelsen, 2013) and the 2012 costs are applied to all years. Remaining costs are retrieved from Kolada. All costs are converted to 2016 EUR prices. | | | | |

**Table of underlying regression output**

| **Full** **regression results**  Table S2: Panel data analysis of the effect of dementia on formal care costs over 10 years (expressed in EUR 2016 prices). | | | |
| --- | --- | --- | --- |
|  | β (Euro) | SE Robust | p |
| Dementia | 3,410 | 750 | <0.001 |
| Years |  |  |  |
| 3 years before (reference) | - |  |  |
| 2 years before | 1,781 | 422 | <0.001 |
| 1 year before | 4,307 | 508 | <0.001 |
| Diagnosis/index | 6,003 | 548 | <0.001 |
| 1 year after | 6,666 | 583 | <0.001 |
| 2 years after | 7,096 | 603 | <0.001 |
| 3 years after | 7,815 | 613 | <0.001 |
| 4 years after | 8,630 | 695 | <0.001 |
| 5 years after | 8,904 | 807 | <0.001 |
| 6 years after | 7,471 | 933 | <0.001 |
| Dementia and years before/after diagnosis |  |  |  |
| Dementia and 3 years before (reference) | - |  |  |
| Dementia and 2 years before | 2,044 | 672 | 0.002 |
| Dementia and 1 year before | 4,612 | 764 | <0.001 |
| Dementia and diagnosis/index | 19,966 | 869 | <0.001 |
| Dementia and 1 year after | 30,695 | 961 | <0.001 |
| Dementia and 2 years after | 36,336 | 992 | <0.001 |
| Dementia and 3 years after | 40,466 | 1,050 | <0.001 |
| Dementia and 4 years after | 42,547 | 1,167 | <0.001 |
| Dementia and 5 years after | 43,632 | 1,475 | <0.001 |
| Dementia and 6 years after | 46,317 | 1,966 | <0.001 |
| Sex (male) | -1,123 | 343 | 0.001 |
| Age at diagnosis/index |  |  |  |
| < 70 yeas (reference) | - |  |  |
| 70 – 89 years | 7,201 | 511 | <0.001 |
| ≥ 90 years | 21,076 | 717 | <0.001 |
| Highest educational degree |  |  |  |
| Compulsory (reference) | - |  |  |
| Upper secondary | -1,111 | 377 | 0.003 |
| Higher education | -1,928 | 460 | <0.001 |
| Missing | -2,032 | 1,351 | 0.133 |
| Foreign background | -2,960 | 537 | <0.001 |
| Married or cohabitant | -11,895 | 341 | <0.001 |
| Number of children |  |  |  |
| None (reference) | - |  |  |
| 1 - 2 | -36 | 512 | 0.945 |
| 3 - 4 | -763 | 564 | 0.176 |
| ≥ 5 | -472 | 998 | 0.636 |
| Elixhauser index | 2,447 | 96 | <0.001 |
| Year |  |  |  |
| 2013 (reference) | - |  |  |
| 2014 | -457 | 210 | 0.030 |
| 2015 | 675 | 320 | 0.035 |
| 2016 | 3,839 | 414 | <0.001 |
| Municipality dummies (n=34) | included |  |  |
| Constant | -5,619 | 2,253 | 0.013 |
| Individuals (observations) |  | 30,678 (106,493) | |
| R-squared |  | 0.27 | |

| **Table S3:** Predicted formal care costs of people with dementia and population controls from 3 years before to 6 years after diagnosis by care component, and each component’s share of costs. Expressed in 2016 Euro prices. | | | | | | | | | | | |
| --- | --- | --- | --- | --- | --- | --- | --- | --- | --- | --- | --- |
|  | | **Years from Diagnosis** | | | | | | | | | |
| **Variable** | | **-3** | **-2** | **-1** | **0** | **1** | **2** | **3** | **4** | **5** | **6** |
| **Healthcare** | *Dementia* | 2,868 | 3,642 | 4,368 | 8,490 | 6,127 | 5,281 | 4,724 | 4,562 | 4,246 | 4,149 |
|  | *% of dementia total* | 34% | 30% | 25% | 25% | 13% | 10% | 8% | 8% | 7% | 7% |
|  | *Control* | 2,793 | 3,225 | 3,449 | 3,933 | 3,978 | 4,041 | 4,326 | 4,209 | 4,470 | 4,557 |
|  | *% of* *control total* | 55% | 47% | 37% | 36% | 34% | 33% | 34% | 31% | 32% | 36% |
|  | *Excess Cost* | 75 | 417 | 919* | 4,556* | 2,150* | 1,241* | 398 | 352 | -224 | -408 |
| **Drugs** | *Dementia* | 606 | 643 | 684 | 770 | 860 | 906 | 959 | 942 | 916 | 964 |
|  | *% of dementia total* | 7% | 5% | 4% | 2% | 2% | 2% | 2% | 2% | 2% | 2% |
|  | *Control* | 452 | 536 | 576 | 611 | 726 | 733 | 754 | 832 | 664 | 681 |
|  | *% of control total* | 9% | 8% | 6% | 6% | 6% | 6% | 6% | 6% | 5% | 5% |
|  | *Excess Cost* | 155 | 106 | 107* | 159* | 134 | 173 | 205* | 110 | 252* | 283* |
| **Institutional Care** | *Dementia* | 2,541 | 3,895 | 6,332 | 12,141 | 23,452 | 30,073 | 34,959 | 38,982 | 40,552 | 43,993 |
|  | *% of dementia total* | 30% | 32% | 36% | 35% | 51% | 58% | 62% | 65% | 66% | 71% |
|  | *Control* | 942 | 1,389 | 2,590 | 3,679 | 4,119 | 4,365 | 4,544 | 4,880 | 5,164 | 4,499 |
|  | *% of control total* | 19% | 20% | 28% | 33% | 35% | 36% | 35% | 36% | 37% | 36% |
|  | *Excess Cost* | 1,599 | 2,506* | 3,743* | 8,462* | 19,332* | 25,708* | 30,415* | 34,102* | 35,388* | 39,494* |
| **Home Service** | *Dementia* | 1,908 | 3,425 | 4,827 | 9,151 | 11,098 | 10,992 | 11,382 | 10,538 | 10,599 | 8,218 |
|  | *% of dementia total* | 22% | 28% | 28% | 27% | 24% | 21% | 20% | 18% | 17% | 13% |
|  | *Control* | 767 | 1,529 | 2,487 | 2,597 | 2,632 | 2,784 | 2,973 | 3,446 | 3,319 | 2,685 |
|  | *% of control total* | 15% | 22% | 27% | 23% | 22% | 23% | 23% | 25% | 24% | 21% |
|  | *Excess Cost* | 1,141 | 1,896* | 2,340* | 6,553* | 8,466* | 8,209* | 8,409* | 7,092* | 7,279* | 5,533* |
| **Day Care** | *Dementia* | 202 | 347 | 467 | 853 | 1,867 | 2,383 | 2,612 | 2,655 | 2,598 | 2,479 |
|  | *% of dementia total* | 2% | 3% | 3% | 2% | 4% | 5% | 5% | 4% | 4% | 4% |
|  | *Control* | 43 | 84 | 92 | 60 | 40 | 34 | 18 | 4 | 7 | -97 |
|  | *% of control total* | 1% | 1% | 1% | 1% | 0% | 0% | 0% | 0% | 0% | -1% |
|  | *Excess Cost* | 159 | 262* | 376* | 793* | 1,827* | 2,349* | 2,594* | 2,652* | 2,591* | 2,576* |
| **Short-Term Respite Care** | *Dementia* | 359 | 359 | 726 | 3,051 | 2,443 | 2,284 | 2,133 | 1,983 | 2,109 | 2,469 |
|  | *% of dementia total* | 4% | 3% | 4% | 9% | 5% | 4% | 4% | 3% | 3% | 4% |
|  | *Control* | 77 | 93 | 186 | 196 | 245 | 214 | 275 | 333 | 353 | 220 |
|  | *% of control total* | 2% | 1% | 2% | 2% | 2% | 2% | 2% | 2% | 3% | 2% |
|  | *Excess Cost* | 281 | 266* | 540* | 2,855* | 2,198* | 2,070* | 1,858* | 1,649* | 1,757* | 2,249* |
| **Note:** Adjusted for year, municipality, sex, age at diagnosis, education, foreign background, number of children, marital status and Elixhauser comorbidity index 5 years prior diagnosis. n is the number of observations. * Statistical significance at the 5 percent level. | | | | | | | | | | | |
